# Progress in Public Utilities

**Published:** 1913-01

**Authors:** 


### Progress in Public Utilities

For most parts of the civilized world, the great moral issues of freedom of conscience and personal action, of arbitrary caste systems, of slavery, of popular education, have been solved. Most of the great questions of policy have either been settled, or have been reduced to minor differences of opinion between opposing principles, each modified so as to approach the other in practical application. For example, the dispute between states' rights and national concentration of power, between protection and free trade, have been reduced to a substantial agreement except as to questions of degree. And neither party in such a dispute would undertake a radical experiment but a cautious extension of theory in one direction, subject to a halt at the first indication of unfavorable result.

Statesmanship and, to an increasing degree, practical politics, are coming to deal with economic problems, with the actual carrying out of principles no longer in dispute, with the extension of fair and honorable methods from theory to practice, in both private and public business. It must not be overlooked that the doing away with great principles such as led to the War of the Revolution, of 1812, of the Civil War and perhaps, also the Spanish War, and the substitution of opinions of minor ethical importance, in political life, is not so much a sign of deterioration as of the accomplishment of moral and religious ideals. It was a grand privilege to write, to lecture, to indure insult and oppression, to sacrifice one's property and life, to wage war and overturn government, with the justification of a great moral issue. But we must not forget the terrible suffering and the horrible moral status of at least a part of society which made such moral issues possible. We can not wish for the persistence of such great issues any more than for the persitence of great epidemics and professional ignorance in order to return to the possibility of medical heroism of the old type instead of having the routine every-day heroism of effective study and practice.

The era of economic political issues is of direct interest to the medical profession. The maintenance and restoration of health, the saving of life, depend less and less on control of epidemics of whose nature we are ignorant, more and more on economic details. The stress of poverty, not so much in the literal privational sense as in the difficulty of securing a steady, dependable outlet of one's industry and the ability to meet demands without hardship and anxiety, is rapidly assuming the importance which bacteria have filled.

About a tenth of all deaths are due to violence, of one kind or another. The control of various businesses, especially mining and manufacturing industries, public and private methods of transportation will eliminate a large share of violent deaths. Suicides are largely due, directly and indirectly, to economic conditions. Homicide is largely controlable by education, especially the education of the public as to the necessity of limiting procreation of criminals. And this education depends largely upon economic principles. A considerable part of the mortality is due to overcrowding in cities and can be remedied only by rational attention to economic details. Whether dealing with the literally overcrowded part of the population or not, another important item in the death rate is the failure to obtain proper sanitation and hygiene in dwellings, shops and other buildings. But, without actual or virtual confiscation of property, this reform cannot be secured until the enormous tax rate of the average city is so reduced by the application of ordinary principles of business economy to municipal affairs, that land lords can afford to provide light, ground space and ideal constructive features and still have a fair margin of profit.

Many acute cases of financial suffering, often leading to suicide, to exposure and overwork of women and children, with resulting disease and death, are due to insecure investments. In our experience, the great majority of instances of loss of savings by our own profession are due to investments in organizations not conducted in good faith. One of the important functions of government, already recognized in various countries and some of our own states, is the control of stock companies so as to secure the interests of active participants, investors and the public buying the commodity or service of the corporation.

Without ignoring various other economic factors, including details of political dispute, as the tariff, control of large industries, etc., the panics and periods of financial depression of recent years have been due, not so much to lack of actual productive wealth, available labor and desire to purchase either material or labor, as to the difficulty of securing the actual cash to effect an exchange. One hundred years ago, with a population mainly dwelling in the country and with each family directly producing most of its own necessities, the per capita cash was five to ten dollars, representing as many day's labor. At present, with the majority of the population depending upon actual cash for the purchase of nearly all of its needs, the per capita cash is thirty to thirty-five dollars, representing about ten days labor, and with various business and political factors tending to concentrate this cash where it cannot be available as a medium of exchange. Thus, with supplies of necessities and convenience in abundance, with men ready and willing to work and to demand these supplies, there are frequently periods of months or years in which the available circulation of money is almost at a stand still. In short, the body politic is in much the condition of a normal animal with the portal vein ligated so that, in spite of a sufficient medium of circulation for the whole body, it practically bleeds to death into its own portal system. And, in a literal, individual sense, as well as a figurative, aggregate sense, this condition leads to suffering, disease and death.

The lessening of infectious disease, is gradually overturning the mortality tables so that, in the future, the more important fatal diseases will be those of degeneration, senile change, and accident. The average length of life has already increased nearly ten years in the last thirty years. It is no kindness to save an infant from the non-specific infection of the long nursing tube, or a youth from consumption, or an adult from typhoid, and to kill him by cerebral apoplexy, diabetes, etc., or to drive him to insanity, crime, homicide or suicide, by economic stress. Everything that conduces to general prosperity, that adds to the ease of exchange of money, that increases the purchasing power of the dollar, is an important factor in health and a worthy object of political consideration.

